# FZD2 promotes TGF-β-induced epithelial-to-mesenchymal transition in breast cancer via activating notch signaling pathway

**DOI:** 10.1186/s12935-021-01866-3

**Published:** 2021-04-08

**Authors:** Dilihumaer Tuluhong, Tao Chen, Jingjie Wang, Huijuan Zeng, Hanjun Li, Wangmu Dunzhu, Qiurong Li, Shaohua Wang

**Affiliations:** 1 Department of General Surgery, Jinling Hospital, Medical School of Nanjing University, No. 305 East Zhongshan Road, Nanjing, 210002 China; 2 Department of Pediatric Surgery, Guangzhou Institute of Pediatrics, Guangdong Provincial Key Laboratory of Research in Structural Birth Defect Disease, Guangzhou Women and Children’s Medical Center, Guangzhou Medical University, Guangzhou, Guangdong China

**Keywords:** Breast cancer, FZD2, Poor prognosis, EMT, Notch signaling pathway

## Abstract

**Background:**

Breast cancer (BC) is one of the commonest female cancers, which is characterized with high incidence. Although treatments have been improved, the prognosis of BC patients in advanced stages remains unsatisfactory. Thus, exploration of the molecular mechanisms underneath BC progression is necessary to find novel therapeutic methods. Frizzled class receptor 2 (FZD2) belongs to Frizzled family, which has been proven to promote cell growth and invasion in various human cancers. The purpose of our current study was to detect the functions of FZD2 in BC and explore its underlying molecular mechanism.

**Methods:**

The level of FZD2 was measured in BC tissues by quantitative real-time polymerase chain reaction (qRT-PCR), western blot, immunohistochemistry (IHC), respectively. Cell Counting Kit-8 (CCK-8), colony formation assay, transwell assays, wound healing assay and flow cytometry analyses were separately conducted to detect cell viability, invasion, migration, apoptosis and cell cycle distribution. The levels of Epithelial-mesenchymal transition (EMT) biomarkers were examined by using Immunofluorescence assay. Xenograft tumorigenicity assay was performed to assess the effect of FZD2 on tumor growth in vivo.

**Results:**

FZD2 mRNA and protein expression was abundant in BC tissues. Moreover, high level of FZD2 had significant correlation with poor prognosis in BC patients. In vitro functional assays revealed that silencing of FZD2 had suppressive effects on BC cell growth, migration and invasion. Animal study further demonstrated that FZD2 silencing inhibited BC cell growth in vivo*.* In addition, FZD2 induced EMT process in BC cells in a transforming growth factor (TGF)-β1-dependent manner. Mechanistically, knockdown of FZD2 led to the inactivation of Notch signaling pathway.

**Conclusion:**

FZD2 facilitates BC progression and promotes TGF-β1-inudced EMT process through activating Notch signaling pathway.

**Supplementary Information:**

The online version contains supplementary material available at 10.1186/s12935-021-01866-3.

## Background

Breast cancer (BC) is one of the commonest life-threatening female cancer types. As reported in 2019, BC accounts for 30% among all new female cancer cases [1]. Local recurrence and distant metastasis are the main reasons for the high mortality of BC patients [2]. Although various treatments have been developed for BC patients, the effective therapeutic targets remain limited. Therefore, exploring the molecular mechanism underneath BC progression is of great significance to develop novel therapeutic strategies.

Frizzled family proteins (FZDs, including FZD1-FZD10) function as cell surface receptors of WNT signaling pathway. Each member of FZDs can activate WNT signaling pathway through interacting with different WNT proteins [3]. As previously reported, FZD2 is dysregulated in various cancer types, such as oral squamous cell carcinoma (OSCC) [4], gastric cancer (GC) [5], endometrial cancer (EC) [6], hepatocellular carcinoma (HCC) [7] and tongue squamous cell carcinoma (TSCC) [8]. There is also a study reported that FZD2 suppresses tumor growth in salivary adenoid cystic carcinoma [9]. In addition, non-canonical FZD2 pathway has been recognized to be a regulator for epithelial-to-mesenchymal transition (EMT) and migration [10]. Nevertheless, the mechanism of FZD2 underneath BC progression remains largely unknown.

EMT process is closely related to metastasis and chemotherapy resistance in human cancers [11]. During EMT process, epithelial cells undergo a variety of biochemical alterations and thus change to mesenchymal phenotypes [12]. EMT is closely correlated with the development of human cancers. For example, EMT progress in bladder cancer leads to the high tumor grades and stages [13]. Alterations on the levels of epithelial or mesenchymal markers contribute to the change of EMT process [14]. The expression of EMT markers is tightly associated with the progression of adenocarcinoma and squamous cell carcinoma [15]. Importantly, expression changes of EMT markers are also correlated with BC progression [16].

TGF-β family is a group of cytokines that can regulate EMT process in cancer progression [17]. For example, TGF-β1 promotes the acquisition of a mesenchymal phenotype and thus promotes cell invasion and migration [18]. Notch signaling pathway primarily exerts functions by controlling cell fate decisions, differentiation, and proliferation [19]. According to previous studies, Notch signaling pathway can be involved in EMT process in several different cancer types, such as lung cancer, pancreatic cancer and breast cancer [20–22].

To summarize, the present study focused on the functions of FZD2 in BC progression and its regulatory effects on TGF-β1-inudced EMT and Notch signaling pathway.

## Materials and methods

### Tissue samples

Primary invasive ductal carcinoma tissues and adjacent normal tissues were obtained from female patients at the Jinling Hospital, affiliated with the Medical School of Nanjing University. All participants didn’t receive neoadjuvant therapies before the operation. 105 paraffin sections of breast cancer samples were used to perform IHC experiment, and 42 pairs of breast cancer and adjacent fresh frozen samples were used for qRT-PCR and western blot analysis. The written informed consent had been collected from each patient before the study. The investigation was approved by the ethics committee of Jinling Hospital.

### Cell culture and treatment

SK-BR-3, MCF-7 and MDA-MB-231 cell lines used in this study were obtained from the Type Culture Collection of the Chinese Academy of Sciences (Shanghai, China). MCF-10A (normal human mammary epithelial cell line) purchased from KeyGEN Biotech Company (Nanjing, China) was used as the control cell line. All cell lines were grown in Dulbecco’s Modified Eagle’s medium (DMEM, KeyGEN) supplemented with 10% fetal bovine serum (A3160801, Gibco) and 1% penicillin–streptomycin. Cell culture was accomplished in a humidified incubator containing 5% CO_2_ at 37 °C. Cell passage was performed when the confluence reached to 90%. Reagents used in this study, including TGF-β1, TGF-β type I Receptor inhibitor SB431542, Notch pathway inhibitor FLI-06, were separately purchased from PeproTech (#100–21, USA), MedChemExpress (HY-10431, USA) and MedChemExpress (HY-15860, USA).

### siRNA Transfection and plasmid construction

siRNAs targeting FZD2 were designed and synthesized by Ribobio (Guangzhou, China). For the silencing of FZD2, siRNAs were transfected into SK-BR-3 and MDA-MB-231 cells in accordance with the instruction manual for Lipofectamine 3000 (#L3000015, Invitrogen, Carlsbad, CA, USA). The full-length cDNA sequence of FZD2 was amplified and inserted into GV141 vector (Genechem, China) for overexpressing FZD2. The levels of FZD2 mRNA and protein were separately checked by qRT-PCR and western blot. Sequences for all siRNAs are as follows:

**Table:** 

si-FZD2-1	CATCCTATCTCAGCTACAA
si-FZD2-2	CCATCATGCCCAACCTTCT
si-FZD2-3	CCCGATGGTTCCATGTTCT

### RNA extraction and qRT-PCR

Total RNA was extracted from tissues with RNA kit (Promega), whereas those extracted from cells were accomplished with TRIzol (Invitrogen). RNA was reverse transcribed into cDNA using PrimeScript RT Master Mix (Perfect Real Time, TaKaRa, Shiga, Japan) and then analyzed by qRT-PCR with SYBR Green Master Mix on an Applied biosystem 7500 machine (USA). 2^−ΔΔCT^ method was used to calculate relative mRNA expression by normalizing to GAPDH. Sequences for all primers used for qRT-PCR were provided in Additional file 1: Table S1.

### Western blot

RIPA buffer (Beyotime) was used for extraction of total protein from tissues and cells. BCA Protein Assay Kit (KeyGen Biotech, Nanjing, China) was applied to measure protein concentration. After resolved on SDS-PAGE, the protein lysates were transferred onto a polyvinylidene fluoride (PVDF) membrane (Immobilon, USA). After blocked in 5% non-fat milk, the membranes were incubated with primary antibodies at 4 °C overnight. After washing, the membranes were incubated with the secondary antibody (1: 5000, Abcam) at 37 °C for 1 h. Primary antibodies used in this experiment were shown in Additional file 1: Table S2. The blots were detected by ECL (NCM Biotech, China) kit.

### Immunohistochemistry (IHC)

For IHC staining, sections were dewaxed with xylene and rehydrated in ethanol. To avoid non-specific staining, samples were washed with PBS and blocked with 5% BSA at room temperature for 30 min. After washing, sections were incubated with primary antibodies against FZD2 (1:50, Abcam), TGF-β1 (1:500, Proteintech), Ki-67 (1:10,000, Proteintech) at 4 °C overnight. Afterwards, sections were incubated with secondary antibody kit SP0031 and/or SP0021 (Solarbio, China) in accordance with the instruction manual. Images were taken under a microscope (Olympus CX41, Japan).

### Evaluation of staining of tissue slides

After immunostaining, FZD2 in different tissues was evaluated by a semi-quantitative immunoreactivity scoring system (IRS). The indexes of Immunostaining intensity were separated as 0 (no immunostaining), 1 (weak), 2 (moderate) and 3 (strong). The scores for immunoreactive cells were separately defined as 0 (no immunoreactive cells), 1 (less than10%), 2 (between 10 and 50%), 3 (between 51 and 80%) and 4 (more than 80%). The IRS index for each case ranges from 0 to 12. FZD2 was considered to be upregulated in cases with 6 or higher IRS score, whereas FZD2 was considered to be downregulated in those with IRS score less than 6.

### Cell counting kit 8 (CCK-8) assay

The transfected cells were plated into 96-well plates at 6–8 × 10^3^ cells per well in 100 μL cell suspension. After added CCK-8 solution (10 μL) (Dojindo, Kumamoto, Japan) at six different time points (0 h, 24 h, 48 h, 72 h, 96 h, 120 h), cells were incubated at 37 °C for 2 h. Finally, a microplate reader was applied to measure the absorbance at 450 nm (OD 450 nm).

### Colony formation assay

The transfected cells were seeded in 6-well plates at 3 × 10^3^ cells per well and cultured in DMEM containing 10% FBS. At day 14, cells were fixed with methanol for 10 min and stained by 0.1% crystal violet for 15 min. After captured the pictures, the number of colonies was recorded manually.

### Cell migration and invasion assays

Matrigel-coated 24-well Transwell chambers (Corning Incorporated, Corning, NY, USA) and non-coated Transwell chambers (#353097; BD Biosciences, USA) were separately used for cell invasion assay and cell migration assay. Twenty-four hours after transfection, the cells were plated into the upper compartment containing serum-free DMEM at a density of 2 × 10^4^ cells per well. The lower chamber was added with DMEM containing 20% FBS. Forty-eight hours later, the cells in the upper chamber were removed with cotton swabs, while the cells on the lower surface were fixed with 4% paraform-aldehyde for 10 min and stained with 0.1% crystal violet for 15 min. The results were obtained using an inverted microscope (Olympus, Japan).

### Wound healing assay

Twenty-four hours after siRNA transfection, cells were seeded in 6-well plates at a density of 1 × 10^6^ cells each well. After cell attachment, a scratch was created with a 200 μL pipette tip in the cell layer. Cells that had been scraped off were washed away with PBS. Forty-eight hours later, the distance of wound was measured by observing images at 0 h and 48 h.

### Flow cytometry analysis of cell cycle distribution

Twenty-four hours after siRNA transfection, cells were plated into 6-well plates at a density of 1 × 10^6^ cells per well. Next, cells were rinsed in prepared phosphate buffer saline (PBS). After incubated with the propidium iodide (KeyGEN, China), a flow cytometer was applied to detect the cell population at different phase (G0/G1, S and G2/M). Percent of cells in different phases was measured by BD FACSCantoII (BD Biosciences, USA).

### Flow cytometry analysis of cell apoptosis

Twenty-four hours after siRNA transfection, EDTA-free trypsin (Beyotime, China) was used for cell digestion, and 1 × 10^6^ cells/ml were counted. Cell apoptosis assay was processed under the FITC Annexin V Apoptosis Detection Kit (BD Biosciences), cell apoptosis was detected by BD FACSCanto II (BD Biosciences).

### Immunofluorescence microscopy

Cells were fixed in parafolmaldehyde (4%) for 20 min and permeabilized with 0.5% Triton X-100 in PBS for 10 min. Cells were blocked with PBS/BSA 5% for 30 min. The membranes were incubated with primary antibody against E-cadherin and Vimentin (1: 50, proteintech) at 4 °C overnight. After washing, the membranes were incubated with green-fluorescence conjugated Affinipure antibody (Coralite 488, diluted 1:100, Proteintech) or Red-fluorescence conjugated Affinipure antibody (Coralite 594, diluted 1:100, Proteintech). After washing, DAPI (Sigma, Cat# D-9542) was used for the nuclei staining. Images were visualized using a Nikon ECLIPSE NI (Japan) microscope.

### Xenograft tumorigenicity assay

For animal study, 12 female BALB/c nude mice (4 weeks old) were bought from the Comparative Medicine Department of Jinling Hospital. MDA-MB-231 cells (1 × 10^7^ cells/ 200μL) were injected into the left rib side. Mice were randomly divided into two groups (n = 6 per group) after the tumor volume reached 100mm^3^. si-FZD2 and si-NC (Ribobio, China) [23, 24]was administered by intratumoral injection at a dose of 5 nM /mouse administered once every 3 days and continuously for 14 times. Forty days after injection, all mice were euthanasia by an intravenous injection of Sodium pento-barbital (100 mg/kg) and the tumors were removed. Tumor tissues were embedded in paraffin for further analyses. Animal study was followed AVMA Guidelines for the euthanasia of Animals (2020 Edition) and the protocol of the animal ethics committee of Jinling Hospital.

### TUNEL assay

Apoptosis in tumor tissues removed from nude mice was detected by using the TUNEL assay kit (Roche, USA) according to the manufacturer’s guidelines.

### Statistical analysis

Statistical significance of differences was analyzed with two-tailed student’s *t* test (between two groups) or one-way/two-way ANOVA (among multiple groups). All experimental data were obtained from three or more independent experiments and shown as mean ± SD (standard deviation). The correlation between FZD2 expression and the clinicopathological characteristics of BC patients was analyzed using the Chi-squared test. SPSS v17.0 (SPSS Inc., Chicago, IL, USA) and Prism v8.0 (GraphPad, San Diego, CA, USA) were utilized for statistical analyses. Data were statistically significant when *p* values less than 0.05.

## Results

### FZD2 is significantly upregulated in BC tissues and associated with poor prognosis

**Fig. 1:**
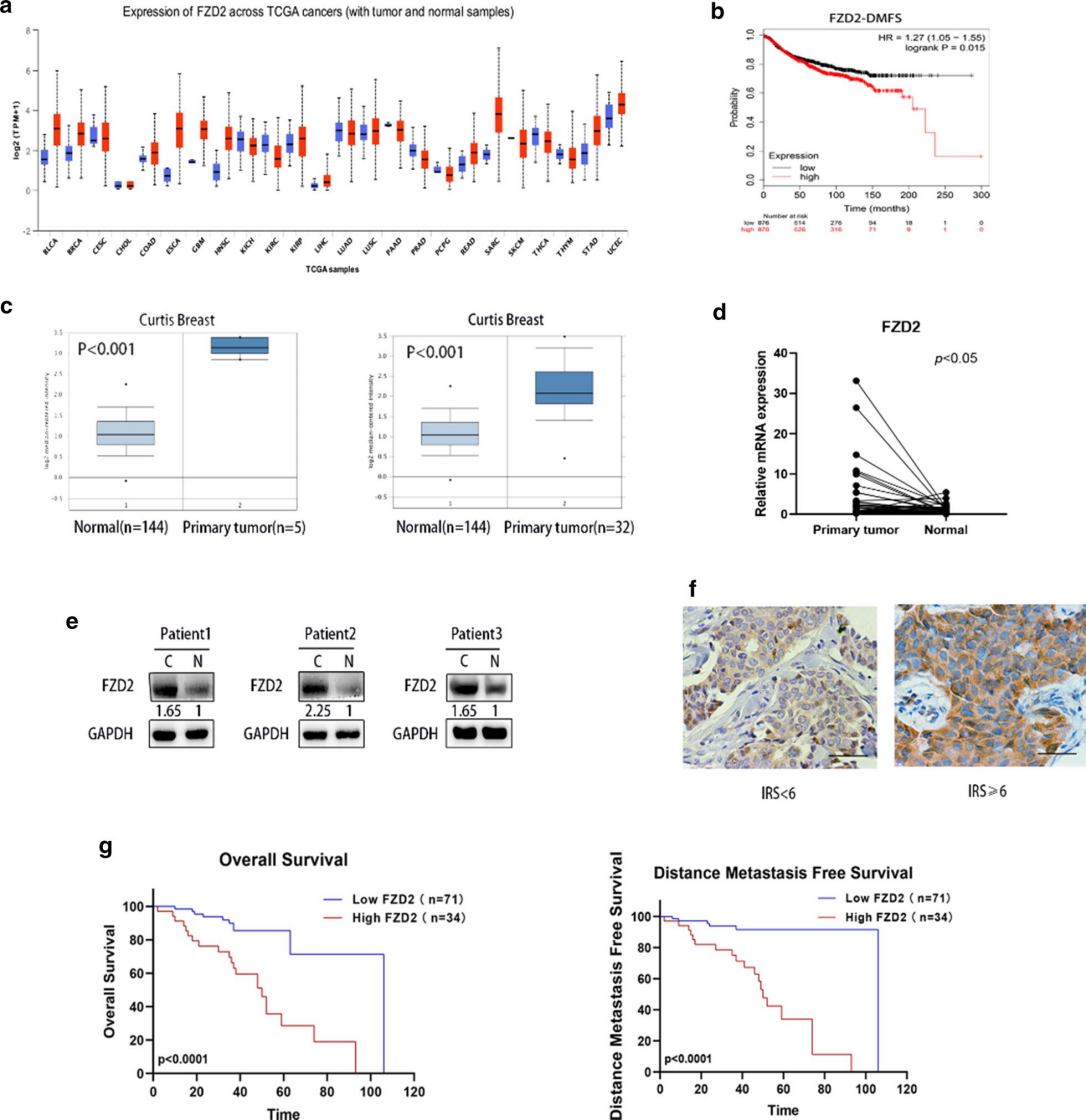
Upregulation of FZD2 in BC tissues is correlated with poor prognosis. **a** Data obtained from TCGA database revealed the FZD2 expression in different types of cancer tissues. **b** DMFS of BC patients with high or low level of FZD2 were analyzed by generating KM plots based on publicly available microarray data. **c** Representative images of FZD2 upregulation in BC tissues were generated from the ONCOMINE database. **d** qRT-PCR detected the mRNA level of FZD2 in 42 BC tissues and adjacent normal tissues. **e** Western blot analysis of FZD2 protein in BC tissues with adjacent normal tissues as controls. **f** Representative images of FZD2 expression in BC tissues were obtained using IHC (Original magnification, × 400, scale bar, 100 μm). **g** OS (left panel) and DMFS (right panel) in BC patients were analyzed by KM method. ^*^p < 0.05, ^**^p < 0.01, ^***^p < 0.001 was a symbol of statistical significance. TCGA, the cancer genome atlas; FZD2, frizzled class receptor 2; BC, breast cancer; KM, Kaplan–Meier; OS, overall survival; RFS, recurrence-free survival; DMFS, distant metastasis free survival

According to UALCAN [25] dataset, FZD2 expression is associated with various tumors (Fig. 1a). Based on TCGA dataset, the prognostic value of FZD2 in 701 BC patients was analyzed. After analysis, FZD2 overexpression was identified to be correlated with worse distant metastasis free survival (DMFS) (Fig. 1b), indicating the potential involvement of FZD2 in cancer progression. Searching from ONCOMINE database (http://www.oncomine.org/resource/login.html), we identified the upregulation of FZD2 in BC tissues (Fig. 1c). Then, we applied qRT-PCR to examine FZD2 mRNA expression in 42 BC tissues relative to adjacent normal tissues. Not surprisingly, FZD2 presented higher expression in BC tissues (Fig. 1d). The protein level of FZD2 exhibited the same tendency in BC tissues, as evidenced by western blot analysis (Fig. 1e). Data of IHC assay revealed that FZD2 was mainly expressed in the cytoplasm (Fig. 1f). Simultaneously, we found that patients with higher FZD2 expression exhibited significantly poorer OS and DMFS than those with lower FZD2 level (Fig. 1g). Finally, we divided the 105 patients into two groups (high FZD2 expression group and low FZD2 expression group). Next, we analyzed the relationship between FZD2 expression and clinical characteristics (Table 1). It was uncovered that overexpression of FZD2 had significant correlations with TNM stages (*p* = 0.006), lymph-node metastasis (*p* = 0.024) and organ metastasis (*p* = 0). Overall, highly expressed FZD2 in BC tissues is correlated with poor prognosis.

**Table 1:** The association between FZD2 expression and clinical-pathological characteristics in breast cancers

Expression of FZD2			
Factors	Low (N = 71)	High (N = 34)	P-value
Age (years)			
≤ 50	35	17	1
> 50	36	17	
Tumor size (cm)			
< 2.5	31	22	0.060
≥ 2.5	40	12	
ER status			
(+)	35	23	0.095
(−)	36	11	
PR status			
(+)	44	20	0.832
(−)	27	14	
Her-2 status			
(+)	22	13	0.511
(−)	49	21	
Ki-67 status (%)			
≤ 20%	24	10	0.824
> 20%	47	24	
Pathological grading			
I–II	51	23	0.655
III	20	11	
TNM stage			
I–II	57	18	0.006*
III–IV	14	16	
Lymph-node metastasis			
pN0	54	18	0.024*
pN+	17	16	
Organ metastasis			
No	65	15	0.000*
Yes	6	19	

*ER* estrogen receptor, *FZD2* frizzled class receptor 2, *Her-2* human epidermal growth factor receptor 2, *PR* progesterone receptor

* P < .05 was considered statistically significant

### FZD2 promotes BC cell growth, migration and invasion, while reduces cell apoptosis in vitro

**Fig. 2:**
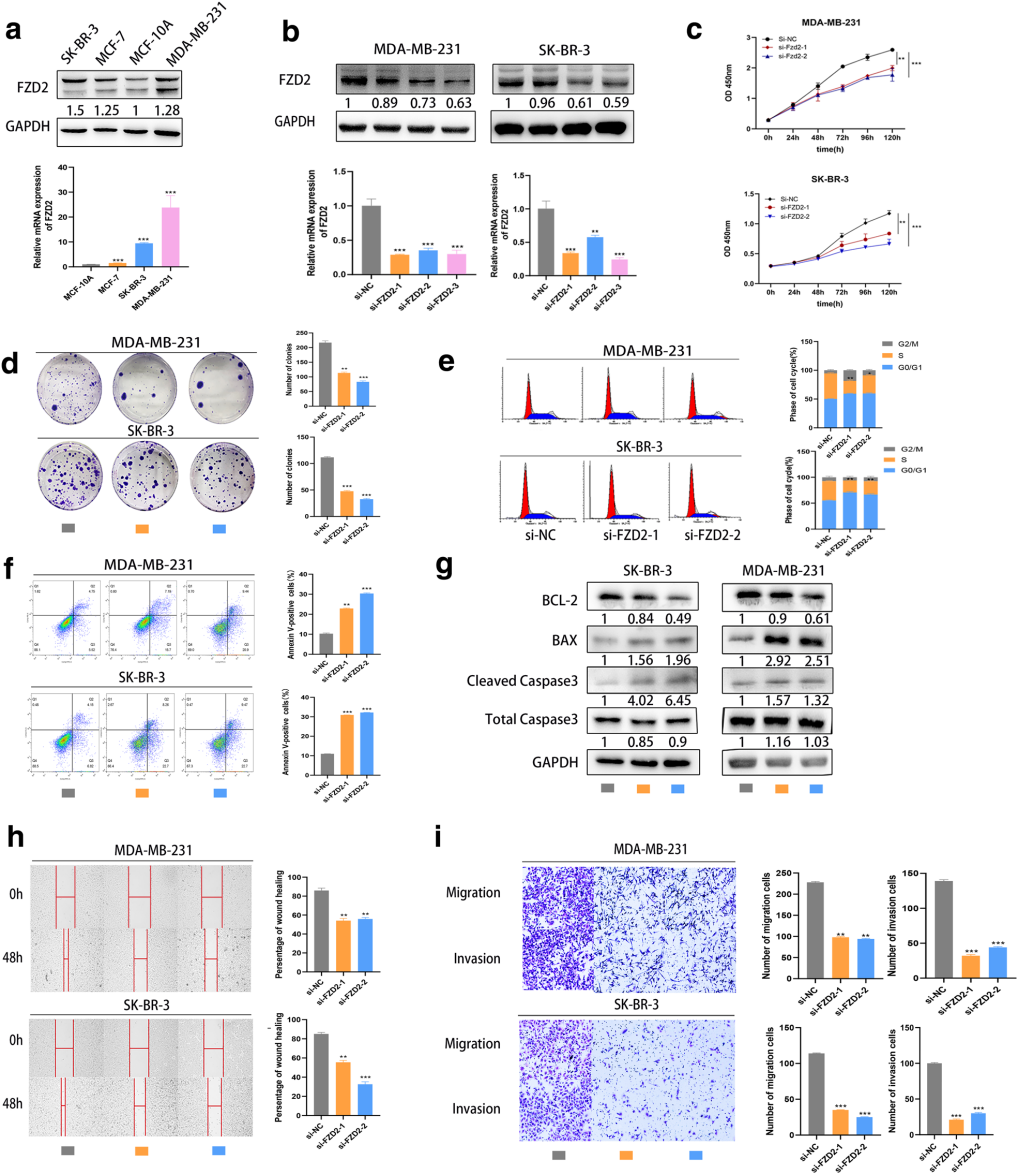
FZD2 is critical for BC cell growth, migration, invasion and apoptosis in vitro. **a** FZD2 mRNA and protein were measured in BC cells with normal MCF-10A cell as control. **b** Knockdown of FZD2 induced by specific siRNAs in MDA-MB-231 and SK-BR-3 cells was identified by qRT-PCR and western blot. **c** CCK-8 assay was implemented to assess cell growth after inhibition of FZD2 expression. **d** The number of colonies was quantified after siRNA transfection in two BC cells. **e** Cell cycle distribution was evaluated by flow cytometry in two BC cells with FZD2 silencing. **f** Flow cytometry analysis of apoptosis in BC cells transfected with si-NC or FZD2-specific siRNAs. **g** Western blot was conducted to detect apoptosis-related proteins in BC cells with FZD2 silencing. **h** Representative images and quantitative bar graphs of wound-healing distance for FZD2-silenced cells. **i** Representative micrographs and quantification of the invaded or migrated cells after FZD2 knockdown. Results were obtained from Matrigel-coated transwell assays and non-Matrigel-coated transwell assays. ^*^p < 0.05, ^**^p < 0.01, ^***^p < 0.001 were symbols of statistical significance. FZD2, frizzled class receptor 2; BC, breast cancer

Furthermore, we investigated FZD2 expression in BC cells through comparing with that in the normal MCF-10A cell. As expected, FZD2 was upregulated in three BC cells (Fig. 2a), among which SK-BR-3 and MDA-MB-231 presented the highest FZD2 level (*p* < 0.01), MCF-7 presented the lowest level FZD2. We thus selected SK-BR-3 and MDA-MB-231 cells for loss-of-function experiments and MCF-7 for gain-of-function experiments. At first, silencing and overexpression of FZD2 were confirmed in indicated cells through qRT-PCR and western blot analyses (Fig. 2b and Additional file 1: Fig. S1A). Since si-FZD2-2 and si-FZD2-3 resulted in the most significant knockdown effect on FZD2, we used these two siRNAs for subsequent experiments. FZD2 silencing dramatically reduced cell proliferation (Fig. 2c, d), whereas overexpression of FZD2 significantly induced cell proliferation (Additional file 1: Fig. S1B, C). Moreover, cells were arrested at G2/M phase after FZD2 knockdown but were arrested at S phase after FZD2 was overexpressed (Fig. 2e and Additional file 1: Fig. S1D). Therefore, we confirmed that FZD2 promotes in vitro cell growth in BC. As for apoptosis, flow cytometry analysis and western blot were conducted in FZD2-silenced or overexpressed BC cells. Then, we observed an enhanced apoptosis rate in cells with FZD2 silencing but a reduced apoptosis rate in cells with FZD2 overexpression (Fig. 2f and Additional file 1: Fig. S1E). Consistently, the changes on the levels of Bax, cleaved caspase 3 and Bcl-2 also presented the negative correlation between FZD2 expression and BC cell apoptosis (Fig. 2g and Additional file 1: Fig. S1F). The mobility of BC cells was evaluated after FZD2 depletion and upregulation. Intriguingly, knockdown of FZD2 weakened the migratory and invasive ability of BC cells (Fig. 2h, i), Overexpression of FZD2 strengthened the migratory and invasive ability of BC cells (Additional file 1: Fig. S1G, H). Accordingly, we identified the important role of FZD2 in regulating BC cell migration, invasion and apoptosis in vitro.

### FZD2 silencing suppresses BC cell growth in vivo

**Fig. 3:**
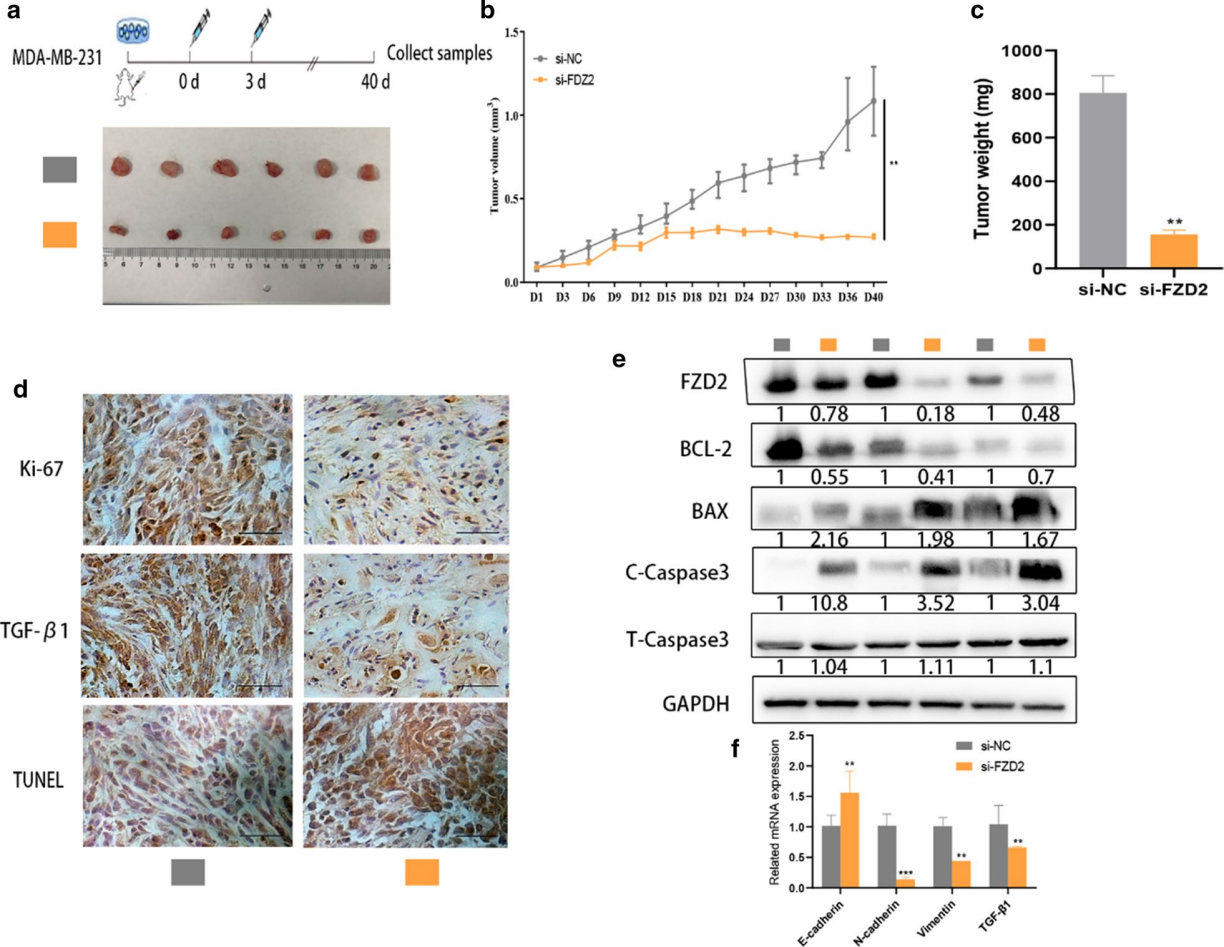
FZD2 promotes BC cell growth in vivo. **a** Images of tumors removed from two groups of mice. **b** Volumes of tumors derived from FZD2-downregulated cells or control cells. **c** Weights of tumors derived from FZD2-downregulated cells or control cells. **d** The positivity of TGF-β1 and Ki-67 in the tumor tissues collected from two groups of mice was detected by IHC (Original magnification, × 400, scale bar = 100 μm). Apoptosis in the tissues collected from two groups of mice was measured by TUNEL assay. **e** Apoptosis-related proteins were detected in two groups of tumor tissues. **f** qRT-PCR analysis of mRNA levels of E-cadherin, N-cadherin, Vimentin and TGF-β1 in tissues obtained from two groups of mice. ^*^p < 0.05, ^**^p < 0.01, ^***^p < 0.001 was a symbol of statistical significance. FZD2, frizzled class receptor 2; BC, breast cancer

Animal models were established to investigate the effect of FZD2 silencing on tumor growth. Forty days after injection, we observed the smaller tumor size in si-FZD2 group than those in si-NC group (Fig. 3a). The volume and weight of tumors in two groups presented the consistent tendencies (Fig. 3b, c). IHC showed that Ki-67 and TGF-β1 positivity was lower in tumor tissues of si-FZD2 group (Fig. 3d). Through TUNEL assays, we confirmed that FZD2 silencing induced apoptosis in vivo (Fig. 3d). In the meantime, the levels of Bax and cleaved caspase 3 (pro-apoptotic proteins) were increased, whereas the level of Bcl-2 (an anti-apoptotic protein) was decreased in response to FZD2 silencing (Fig. 3e). The levels of E-cadherin mRNA were enhanced by FZD2 knockdown, whereas N-cadherin, Vimentin and TGF-β1 were remarkably downregulated after silencing of FZD2 (Fig. 3f). Taken together, FZD2 silencing inhibits BC cell growth in vivo**.**

### Depletion of FZD2 reverses TGF-β1 induced EMT process

**Fig. 4:**
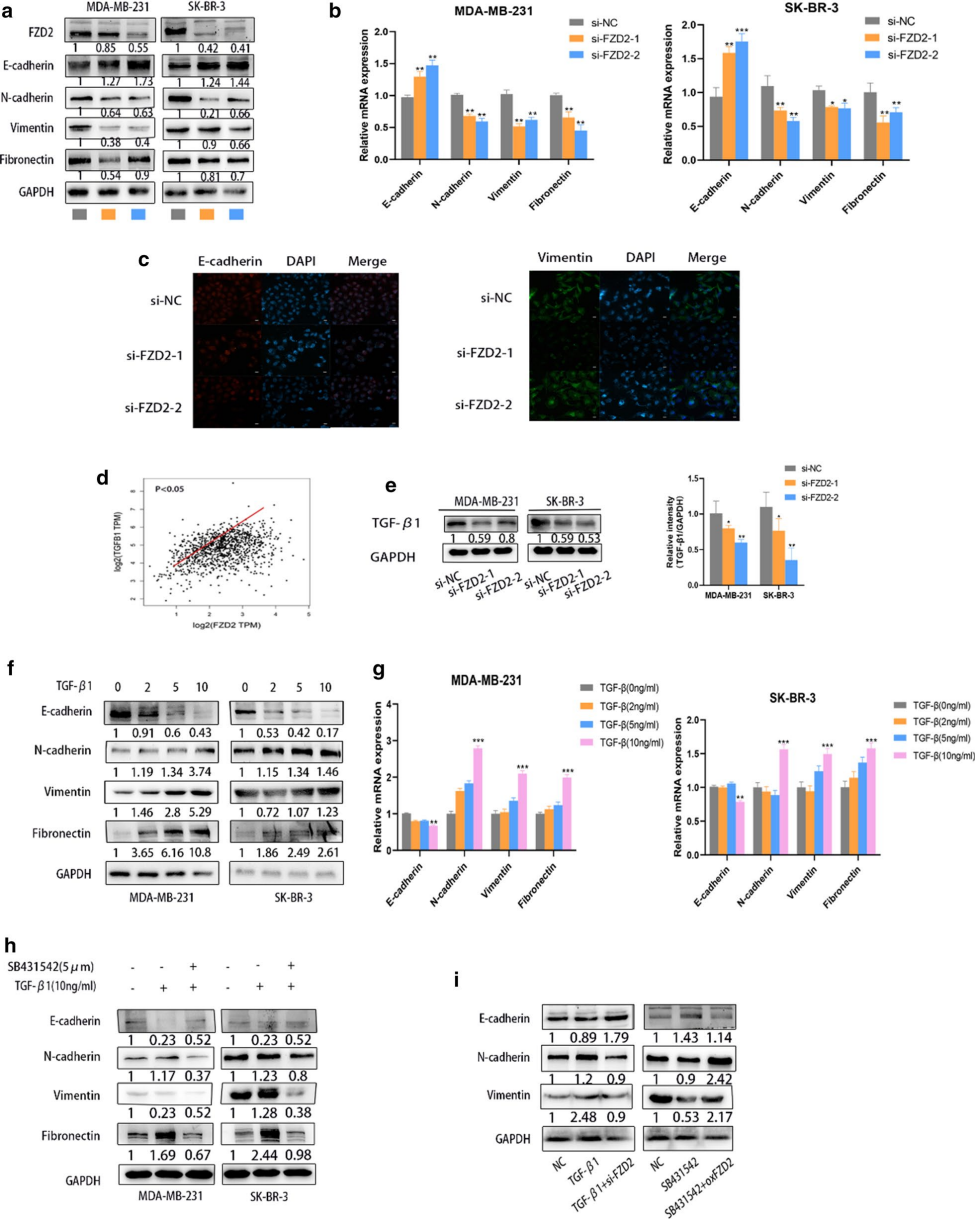
FZD2 induces EMT at TGF-β1 related manner. **a** EMT markers (E-cadherin, N-cadherin, Vimentin and Fibronectin) were examined by western blot in indicated BC cells. **b** EMT markers were detected by qRT-PCR at mRNA levels. **c** The intensity of E-cadherin and Vimentin was tested by immunofluorescence. **d** Expression correlation of TGF-β1 with FZD2 in BC patient samples was analyzed based on GEPIA database. **e** The mRNA and protein level of TGF-β1 was measured in BC cells transfected with si-NC or FZD2-specific siRNAs. **f** EMT markers (E-cadherin, N-cadherin, Vimentin and Fibronectin) were tested in BC cells treated with TGF-β1 (0, 2, 5 and 10 ng/ml) for 24 h. **g** qRT-PCR was used to analyze the mRNA levels of EMT markers in TGF-β1-strengthened BC cells. **h** The levels of related proteins (E-cadherin, N-cadherin, Vimentin, Fibronectin) were measured by western blot in BC cells pre-treated with SB431542 (5 μM) for 2 h, followed by TGF-β1 (10 ng/ml) incubation for another 24 h. **i** The levels of related proteins (E-cadherin, N-cadherin, Vimentin, Fibronectin) were measured by western blot in BC cells pre-treated with TGF-β1 (10 ng/ml) or SB431542 (10 ng/ml) for 24 h, followed by siFZD2 or oxFZD2 incubation for another 48 h. ^*^p < 0.05, ^**^p < 0.01, ^***^p < 0.001 was a symbol of statistical significance. FZD2, frizzled class receptor 2; BC, breast cancer; EMT, epithelial-to mesenchymal transition

To analyze whether FZD2 had the potential to affect EMT process, the expression of EMT markers (E-cadherin, N-cadherin, Vimentin and Fibronectin) was assessed in FZD2-silenced cells. As presented in Fig. 4a, b, the mRNA and protein levels of E-cadherin were enhanced by FZD2 knockdown, whereas that of N-cadherin, Vimentin and Fibronectin were remarkably downregulated after silencing of FZD2 (Fig. 4a, b). In addition, Immunofluorescence analysis further revealed that the level of E-cadherin was increased but the level of N-cadherin was decreased in cells with FZD2 knockdown (Fig. 4c). TGF-β signaling plays an essential role in inducing EMT in different types of tumors. According to the data of GEPIA database, TGF-β1 had positive expression correlation with FZD2 in BC patient samples (Fig. 4d). Moreover, the level of TGF-β1 protein was decreased in FZD2-silenced cells (Fig. 4e). Subsequently, the levels of Fibronectin, N-cadherin and Vimentin were significantly increased by the strengthened TGF-β1 in BC cells, whereas the levels of E-cadherin were remarkably decreased (Fig. 4f, g). In addition, TGF-β1-induced protein levels of Fibronectin, N-cadherin and Vimentin were reduced again by introducing TGF-β receptor antagonist SB431542 (Fig. 4h). Through rescue experiment, we determined that knockdown of FZD2 restored TGF-β1-mediated EMT process (Fig. 4i). It was a solid evidence that FZD2 induces TGF-β1-mediated EMT process in BC cells.

### FZD2 positively regulates TGF-β1-induced EMT in BC cells through Notch signaling pathway

**Fig. 5:**
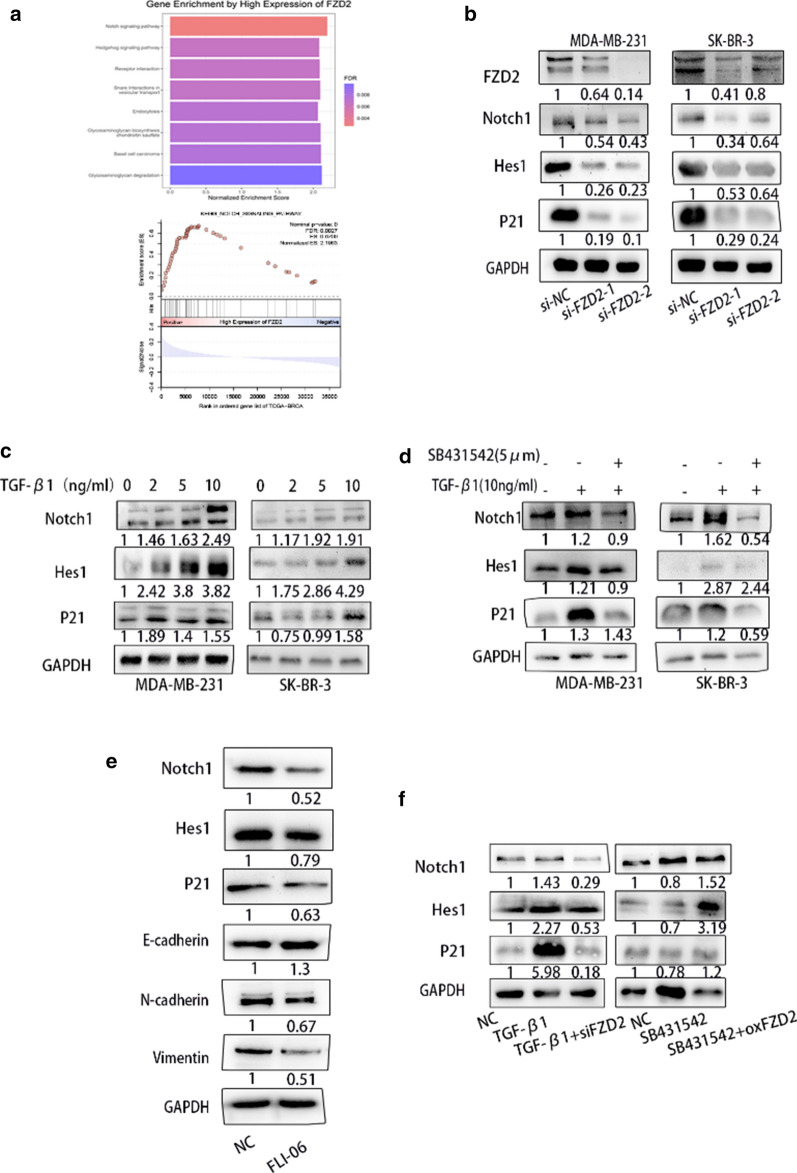
FZD2 positively regulates TGF-β1 expression in BC cells via Notch signaling pathway. **a** The correlation between FZD2 expression and Notch signaling pathway was analyzed based on GESA database. **b** The protein level of FZD2 was measured in BC cells transfected with si-NC or FZD2-specific siRNAs for 72 h. Notch pathway factors (Notch1, P21 and Hes1) were examined by western blot in indicated BC cells. **c** Notch pathway factors (Notch1, P21 and Hes1) were tested in BC cells treated with TGF-β1 (0, 2, 5 and 10 ng/ml) for 24 h. **d** The levels of related proteins (Notch1, Hes1, P21) were measured by western blot in BC cells pre-treated with SB431542 (5 μM) for 2 h, followed by TGF-β1 (10 ng/ml) incubation for another 24 h. **e** The levels of related proteins (Notch1, Hes1, P21, E-cadherin, N-cadherin, Vimentin, Fibronectin) were measured by western blot in BC cells treated with FLI-06 (50 μm). **f** The levels of related proteins (Notch1, Hes1, P21) were measured by western blot in BC cells pre-treated with TGF-β1 (10 ng/ml) or SB431542 (10 ng/ml) for 24 h, followed by siFZD2 or oxFZD2 incubation for another 48 h. ^*^p < 0.05, ^**^p < 0.01, ^***^p < 0.001 was a symbol of statistical significance. FZD2, frizzled class receptor 2; BC, breast cancer; EMT, epithelial-to mesenchymal transition, TGF-β1, transforming growth factor-β1

GSEA database illustrated that FZD2 may regulate the Notch signaling pathway (Fig. 5a). Then, we measured the levels of Notch pathway-related molecules (Notch1, P21 and Hes1). Intriguingly, FZD2 depletion led to the downregulation of these three proteins (Fig. 5b). Meanwhile, the levels of Notch signaling pathway-related proteins were increased after TGF-β1 treatment but were reduced after TGF-β receptor antagonist in BC cells (Fig. 5c, d). Since we have proven that FZD2 induced TGF-β1-mediated EMT process, we thought Notch pathway may be involved in TGF-β1-induced EMT pathway. As presented in Fig. 5e, FLI-06 (NOTCH signaling depressor) treatment led to retarded NOTCH1 signaling since the levels of NOTCH1, Hes1, P21 were distinctly declined. More importantly, FZD2 also restored Notch pathway related protein after TGF-β1 and SB431542 treatment (Fig. 5f). These findings illustrated that FZD2 promotes BC progression and is involved in TGF-β-induced EMT through activating Notch signaling pathway.

## Discussion

Accumulating studies have demonstrated that FZDs can involve in the canonical or non‐canonical WNT pathway [26]. Notably, FZDs can also be intertwined with other signaling cascades. For example, FZD2 can function as a regulator in EMT and metastasis through Fyn/Stat3 pathway [10]. According to previous studies, FZDs can exert various biological functions in different human cancers. For example, FZD1 silencing induces a strong decrease of multidrug resistance protein 1 (MDR1) expression to enhance drug resistance in neuroblastoma (NB) [27]. FZD3 protein expression has close association with the progression of colorectal cancer [28]. High FZD8 expression in human BC tissues is correlated with lymph node metastasis [29]. In our study, FZD2 was identified to be expressed in BC tissues at a significant high level. Importantly, we analyzed the significant correlation between high FZD2 level and the poor prognosis of BC patients. Functionally, FZD2 knockdown led to the inhibition on BC cell growth. Furthermore, silencing of FZD2 had suppressive effects on both migration and invasion of BC cells. Therefore, we confirmed that FZD2 exerts oncogenic role in BC.

Recently, studies have revealed the involvement of EMT in promoting the malignant development of human tumors [30–32]. To date, evidence has indicated that EMT phenomenon favors the distant metastases in epithelial cancers, including breast cancer [33]. FZDs have also been reported as regulators for EMT process in human cancers. For instance, FZD4 promotes the formation of EMT phenotypes in prostate cancer [34]. Sex determining region Y-box 8 (SOX8) and SOX9 induced upregulation of FZD7 regulates EMT in TSCC [35] and HCC [36]. In the current study, FZD2 was verified to be a positive regulator for the EMT process of BC cells. Accumulating studies have suggested that TGF-β promotes metastasis in human cancers, including BC [37]. In the present study, we determined that FZD2 positively regulated TGF-β1 expression in BC cells. Furthermore, TGF-β1 promoted EMT process in BC cells, whereas the effect could be reversed by the suppression of TGF-β1 caused by SB431542, knockdown of FZD2 restored TGF-β1-mediated EMT process critically. The positive expression correlation between TGF-β1 and FZD2 was verified in clinical BC samples. Hereto, we confirmed that FZD2 induces EMT in BC in a TGF-β1-dependent manner.

Notch signaling pathway plays an important role in cancer progression [38]. Importantly, Notch signaling pathway is also known as a modulator for EMT process in several different cancer types [21, 39]. In this work, we found that FZD2 may contribute to BC development by modulating Notch signaling pathway.

The ability to effectively deal with DNA damage is often lost in many types of cancers. Drugs that target this pathway have been clinically validated in patient subgroups, such as the use of PARP (poly ADP-ribose polymerase) inhibitors to treat BRCA1/BRCA2 mutant breast cancer patients [40]. In order to improve the therapeutic effect, DNA damage response (DDR) inhibitors can be used in combination with drugs that target other DDR proteins or signaling pathways [41, 42]. As an oncogene, FZD2 can promote tumor cell proliferation, migration and metastasis. OMP-18R5 (vantictumab) is a FZD family inhibitor. In a phase 1b clinical trial, 48 breast cancer patients were recruited with an overall response rate of 31.3% and a clinical benefit rate of 68.8% [43]. Therefore, as our continuously deepen understanding of the interaction between DNA damage, DDR and immune response, we hope that its use in combination with DDR inhibitors and/or radiation (as sensitizers) may improve the clinical efficacy of immunotherapy. By hindering DNA repair, DDR inhibitors are ideal drugs for combination therapy, which can improve the efficacy of radiation, chemotherapy and immunotherapy.

In conclusion, our findings revealed that FZD2 plays an oncogenic role in BC progression. Mechanistically, FZD2 regulates TGF-β1 signaling and Notch signaling pathway. Our research findings may help to provide an effective therapeutic target for BC patients.

## Supplementary Information

**Additional file 1.** Additional tables and figures.

## Data Availability

The datasets used and/or analysed during the current study are available from the corresponding author on reasonable request.

## Abbreviations

BC: Breast cancer
EMT: Epithelial–mesenchymal transition
FZD: Frizzled class receptor
IHC: Immunohistochemistry
TGF-β1: Transforming growth factor-β
