# Supplementary material for: FZD2 promotes TGF-β-induced epithelial-to-mesenchymal transition in breast cancer via activating notch signaling pathway
Source: Cancer Cell Int. 2021 Apr 8;21:199. doi: 10.1186/s12935-021-01866-3 (PMC8033683; doi:10.1186/s12935-021-01866-3)
Supplement: Supplementary file 1 — Additional file 1. Additional tables and figures. [file 12935_2021_1866_MOESM1_ESM.docx]

**
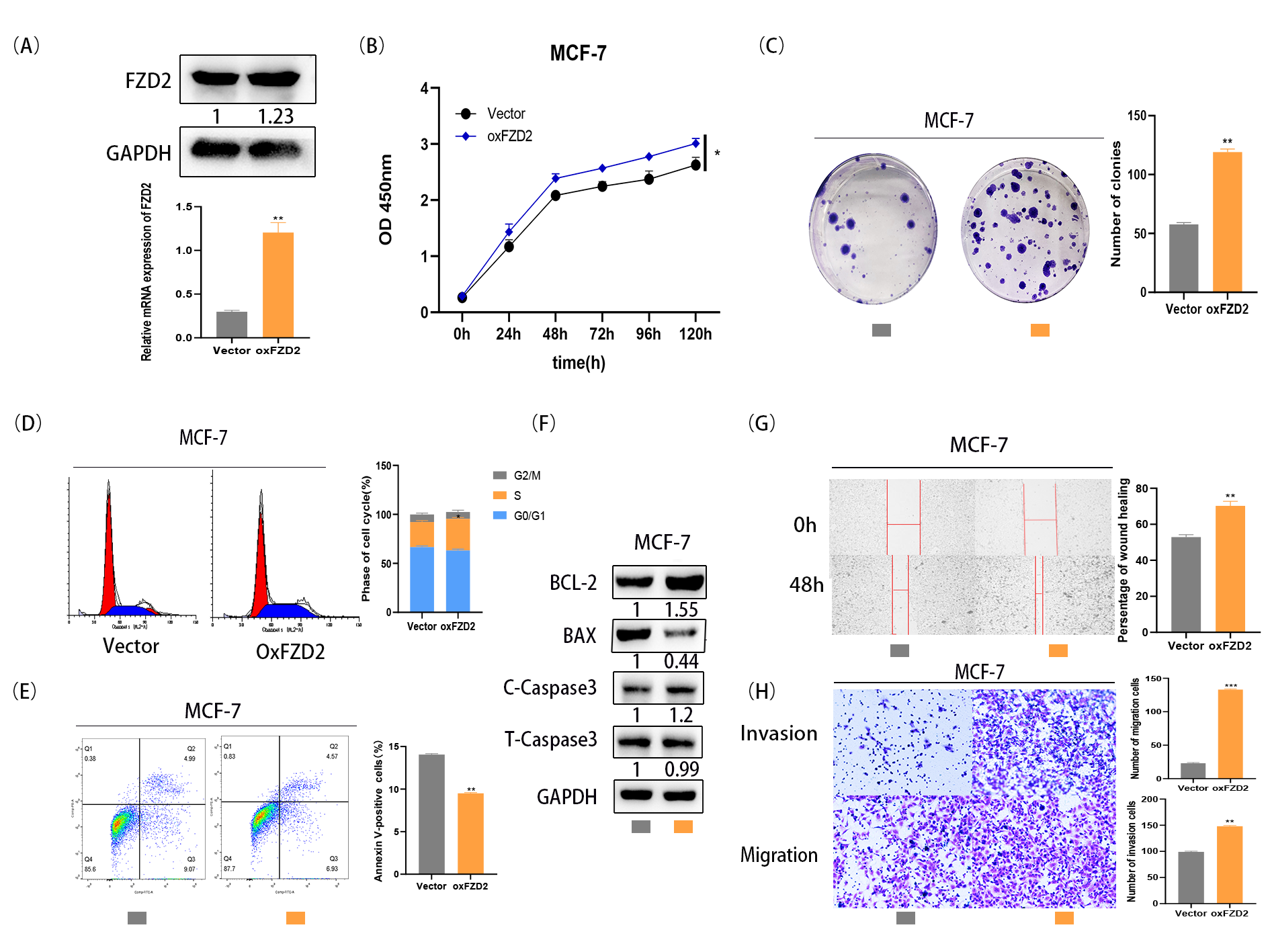
**

**Supplementary Fig.1.** Upregulation of FZD2 is critical for BC cell cell growth, migration, invasion and apoptosis *in vitro*. (A)Overexpression of FZD2 in MCF-7 cells was identified by qRT-PCR and western blot. (B)CCK-8 assay was implemented to assess cell growth after overexpress of FZD2 expression. (C)The number of colonies was quantified after FZD2 overexpression in MCF-7 cells. (D)Cell cycle distribution was evaluated by flow cytometry in MCF-7 cells with FZD2 overexpression. (E)Flow cytometry analysis of apoptosis in BC cells transfected with vector or FZD2-specific cDNA. (F)Western blot was conducted to detect apoptosis-related proteins in BC cells with FZD2 overexpressing. (G)Representative images and quantitative bar graphs of wound-healing distance for FZD2-overexpressed cells. (H)Representative micrographs and quantiﬁcation of the invaded or migrated cells after overexpression of FZD2. Results were obtained from Matrigel-coated transwell assays and non-Matrigel-coated transwell assays.^*^*p* < 0.05, ^**^*p* < 0.01, ^***^*p* < 0.001 were symbols of statistical significance. FZD2, frizzled class receptor 2; BC, breast cancer.


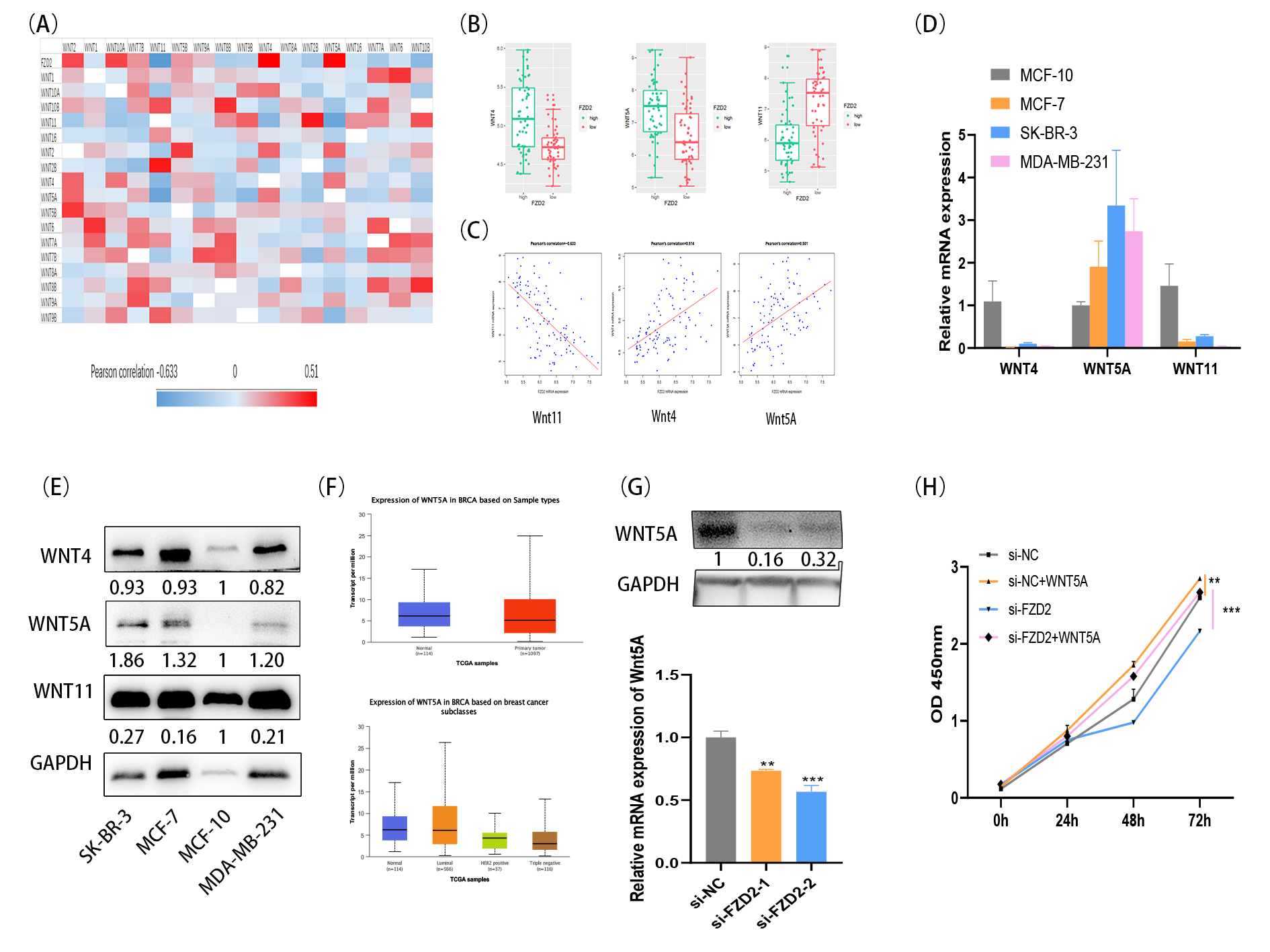


**Supplementary Fig.2.** Wnt5A functions as ligand for FZD2. (A)GSE120129 database interrogated for expression of FZD2 and related mRNA level. Correlation between two genes was analyzed by Pearson statistics. (B)Wnt4 and Wnt5A was overexpressed in FZD2 high level breast cancer, while Wnt11 was decreased. (C)Wnt4 and Wnt5A was positively corelated with FZD2, and Wnt11 was negatively corelated to FZD2. Expression of Wnt4, Wnt5A and Wnt11 was detected by qRT-PCR(D) and Western blot(E) in different breast cancer cell lines. (F)Wnt5A expression in breast cancer according to the online database. (G)Expression of FZD2 and Wnt5A was detected after transfected with si-NC and si-FZD2. (H)Cells were stimulated with recombinant Wnt5A (1ug/ml) after transfected with si-NC or si-FZD2. CCK-8 assay was implemented to assess cell growth after Wnt5A addition.


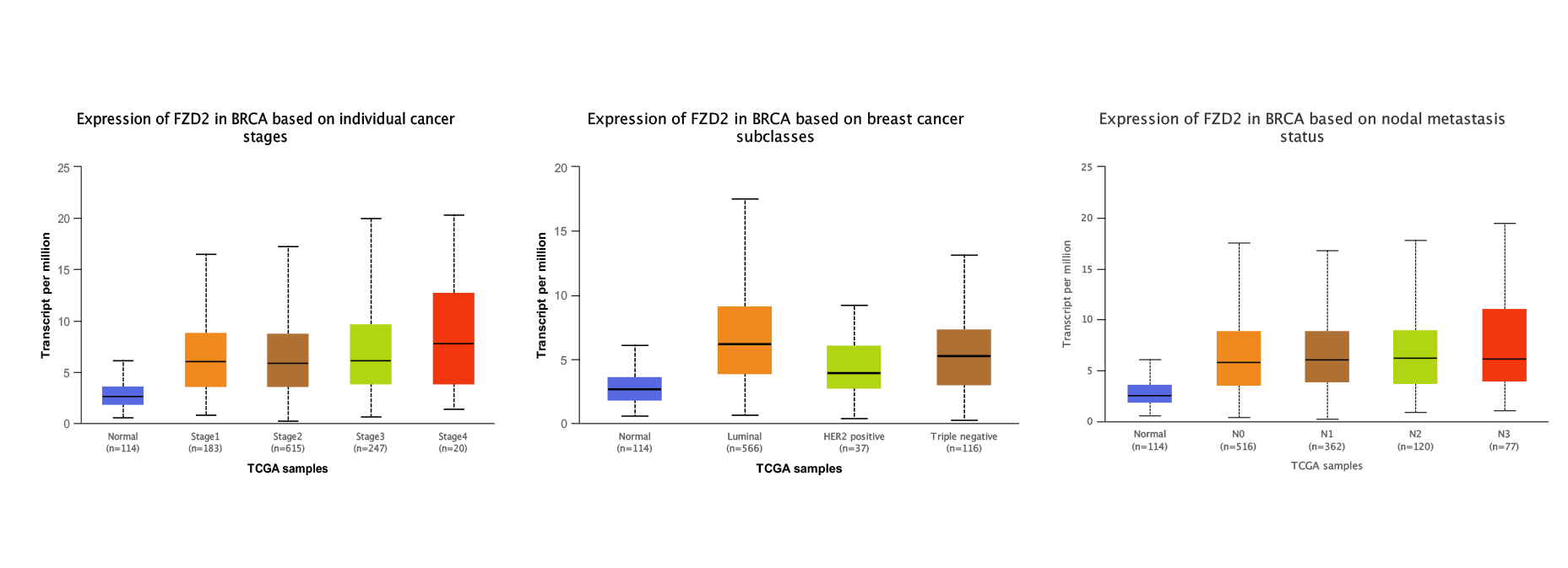


**Supplementary Fig.3.** Expression information about FZD2 according to the online database. (A) Expression of FZD2 in breast cancer subclasses. (B) Expression of FZD2 in individual cancer stages of breast cancer. (C) Expression of FZD2 in different nodal metastasis status of breast cancer.


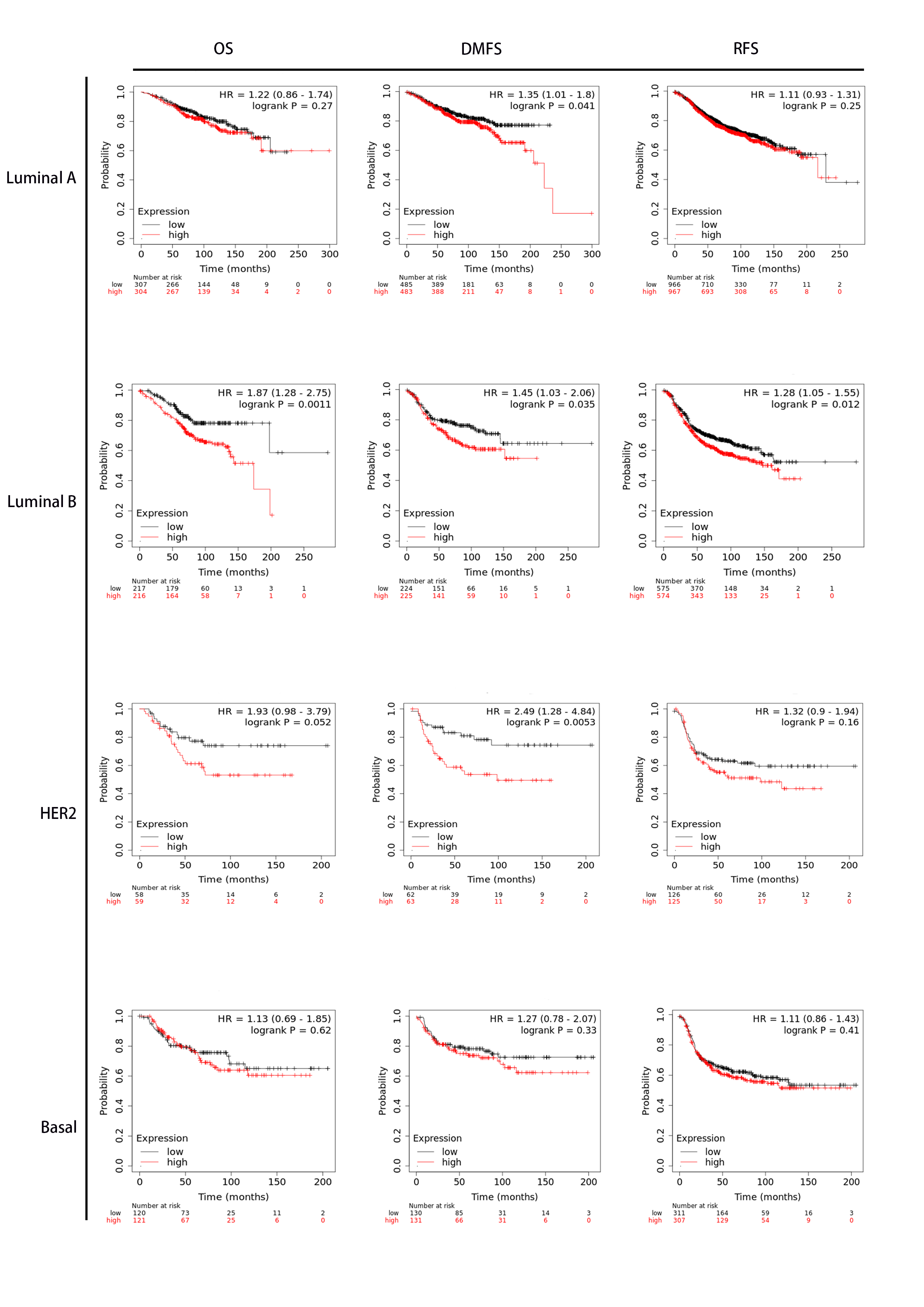


**Supplementary Fig.4.** The overall survival rate, relapse free survival rate and distant metastasis free

survival rate with different types breast cancer patients were analyzed.


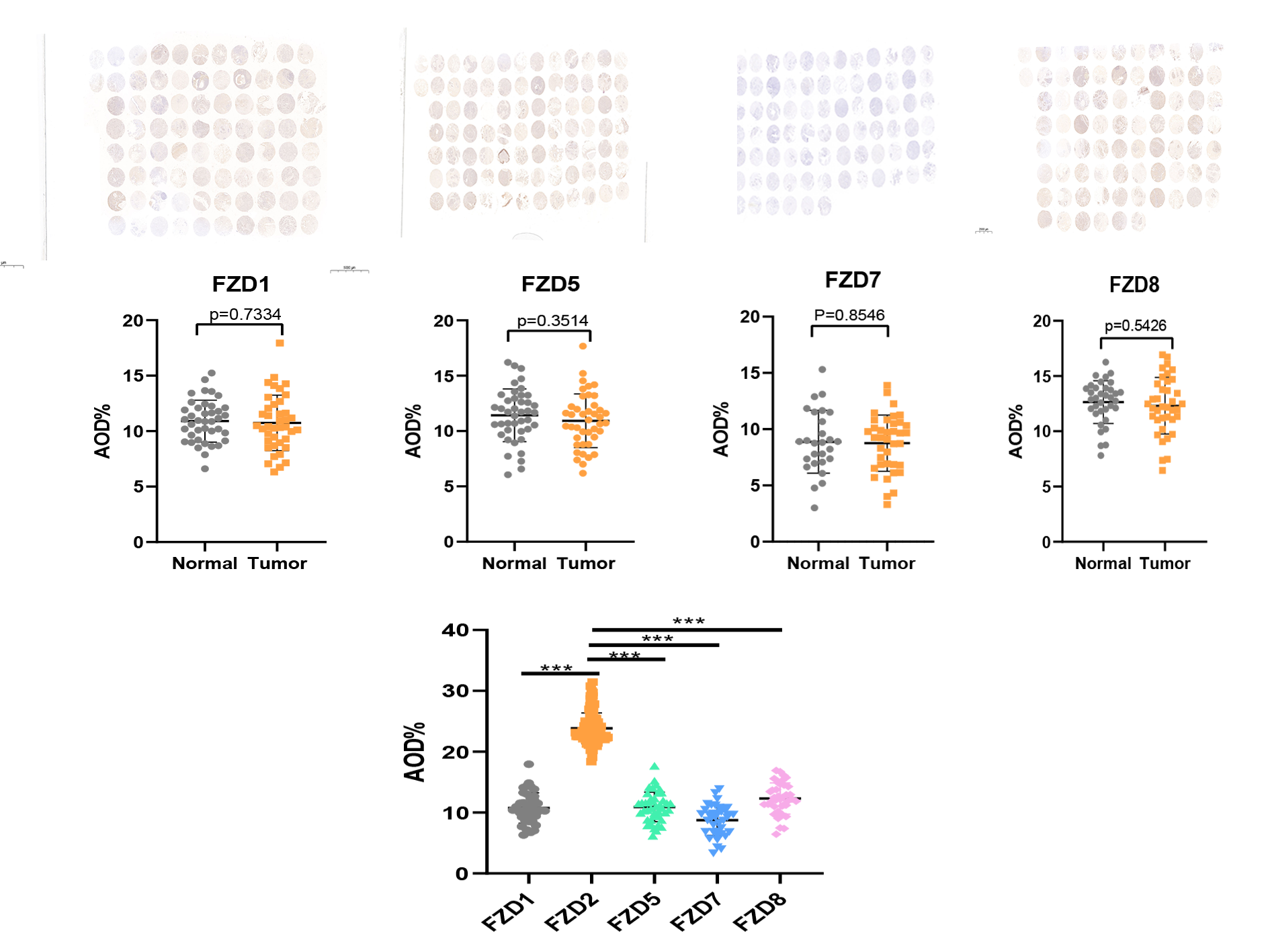


**Supplementary Fig.5.** Expression of FZD1,5,7,8 expression level of breast cancer and adjacant tissues.


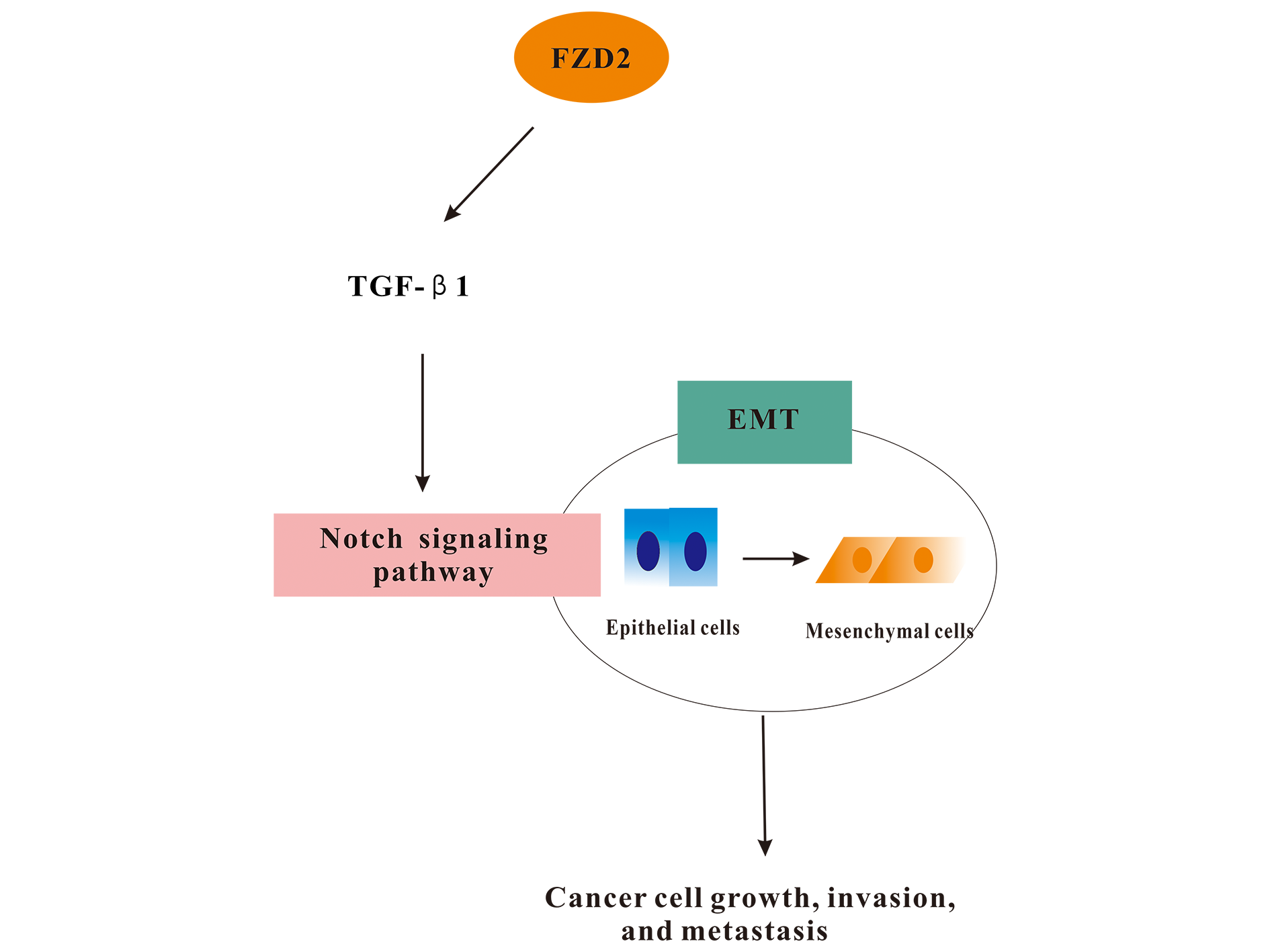


**Supplementary Fig.6.** FZD2 regulates BC progression and EMT process through modulating TGF-β1 signaling and Notch signaling pathway.

**Supplementary Table 1** Primers for qRT-PCR

| E-cadherin | Forward:5'-ATGCCGCCATCGCTTACAC-3' |
| --- | --- |
|  | Reverese:5'-GGTGACCACACTGATGACTCCTG-3' |
| Fibronectin | Forward:5'-TGCCAACCTTTACAGACCTATCC-3' |
|  | Reverese:5'-GCTGACTCGGAGTCTCAGTGATA-3' |
| FZD2 | Forward:5'-ATCTCTGGCGTTCAGGTTAGCA-3' |
|  | Reverese:5'-CCACCCGTCTTTATCACTTTTTG-3' |
| GAPDH | Forward:5'-GCACCGTCAAGGCTGAGAAC-3' |
|  | Reverese:5'-TGGTGAAGACGCCAGTGGA-3' |
| N-cadherin | Forward:5'-CGGAGATCCTACTGGACGGT-3' |
|  | Reverese:5'-CCTTGGCTAATGGCACTTGAT-3' |
| TGF-β1 | Forward:5'-CCCACAACGAAATCTATGACAAG-3' |
|  | Reverese:5'-AGAGCAACACGGGTTCAGGT-3' |
| Vimentin | Forward:5'-CTGGATTCACTCCCTCTGGTT-3' |
|  | Reverese:5'-TCGTGATGCTGAGAAGTTTCGTT-3' |

**Supplementary Table 2** Primary antibodies for western blot analysis

| Name | Company | Catalog Number | Dilution |
| --- | --- | --- | --- |
| BAX | Proteintech | 50599-2-Ig | 1:4000 |
| BCL2 | Proteintech | 12789-1-AP | 1:1000 |
| Caspase3 | CST | #9662 | 1:1000 |
| E-cadherin | Proteintech | 20874-1-AP | 1:5000 |
| FZD2 | Proteintech | 24272-1-AP | 1:500 |
| Fibronectin | Proteintech | 15613-1-AP | 1:500 |
| GAPDH | CST | #2118 | 1:1000 |
| Hes1 | CST | #11988 | 1:1000 |
| Notch1 | CST | #3608 | 1:1000 |
| N-cadherin | Proteintech | 22018-1-AP | 1:2000 |
| P21 | Proteintech | 10355-1-AP | 1:500 |
| TGF-β | Proteintech | 21898-1-AP | 1:1000 |
| Vimentin | Proteintech | 10366-1-AP | 1:3000 |
